# Supplementary material for: Capture of heavy hydrogen isotopes in a metal-organic framework with active Cu(I) sites
Source: Nat Commun. 2017 Mar 6;8:14496. doi: 10.1038/ncomms14496 (PMC5343471; doi:10.1038/ncomms14496)
Supplement: Supplementary Information — Supplementary Figures, Supplementary Tables, Supplementary Notes and Supplementary References [file ncomms14496-s1.pdf]

Supplementary Information

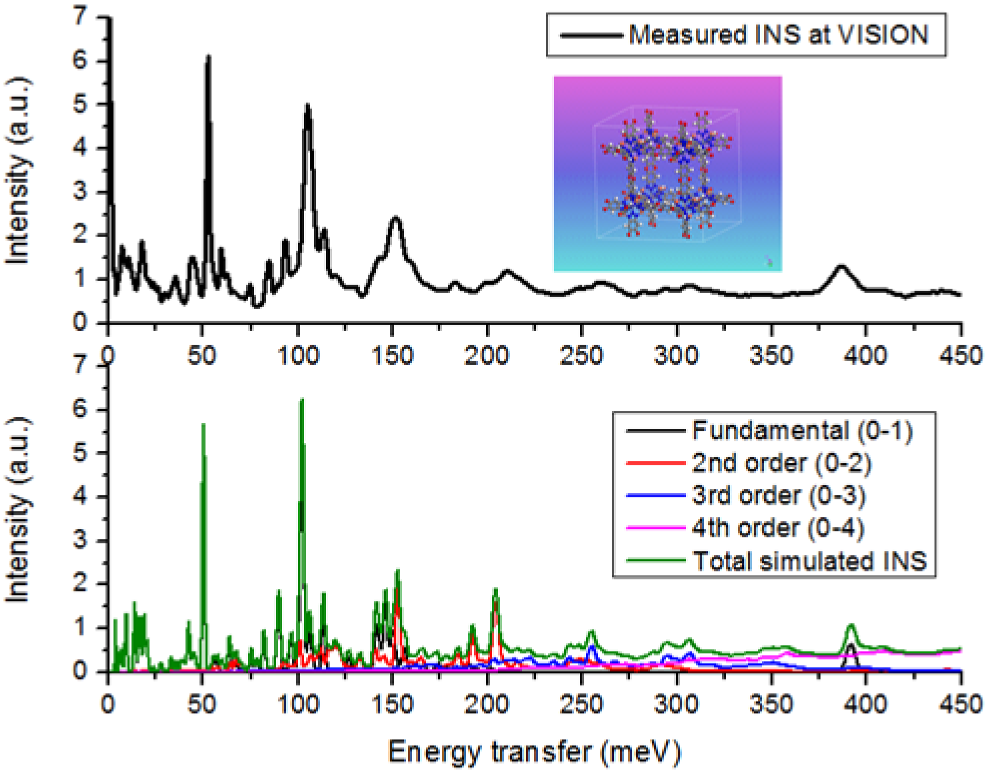

Supplementary Figure 1: Structure validation of Cu(I)-MFU-4*l* by Inelastic Neutron Scattering (INS). Measured (upper panel) and simulated (lower panel) INS spectrum of the blank Cu(I)-MFU-4*l* MOF. The excellent match allows assignment of the peaks and analysis of the corresponding vibrational modes. The vibrational spectrum of the blank MOF was measured by INS as a background. The spectrum itself contains important information about the structure and dynamics of the material. To better understand and interpret the INS spectrum, atomistic calculations have become an important tool in neutron spectroscopy.[1] Specifically, DFT-based ab initio simulation can be used to solve the vibrational frequencies and vectors, which can be further used to calculate the INS. Comparison with the measured INS spectra will then allow us to assign the observed peaks to the corresponding modes. This is an important validation for understanding the structure and dynamics of a material. In this case, vibrational modes have been calculated using CASTEP (PBE, ultrasoft pseudopotentials).[2] The crystal structure is cubic ($Fm\bar{3}m$) with a lattice constant of 30.922 Å, and the primitive cell with 154 atoms was used for the simulation. The SCF tolerance for the electronic structure calculations was 5 10^{-10} eV, and the energy tolerance for ionic relaxation was 5×10^{-9} eV. The tolerance for the interatomic forces was 3 meV Å^{-1}. After convergence was reached, the force constants and the dynamical matrix were obtained using the finite displacement method, from which the phonon frequencies and vibrational modes were calculated. The electronic structure calculations and the vibrations were calculated at the Γ point only. The aClimax software[3] was used to convert the DFT-calculated vibrations to the simulated INS spectra.

Supplementary Note 1: Heat of adsorption. The isosteric heat of adsorption for H_2 and D_2 was independently determined by two experimental techniques, i.e., gas adsorption isotherms and thermal desorption spectroscopy (TDS). Furthermore, the heat of adsorption for H_2, D_2 and T_2 has been obtained by DFT-D3 calculations. Adsorption isotherms of H_2 and D_2 have been measured in the low-pressure region at four different temperatures, i.e. 173, 183, 193, and 203 K (Supplementary Figure 2). The heat of adsorption was determined by two different methods, i.e., the isosteric method and an analytical approach based on the Langmuir model (a detailed comparison between these methods is given elsewhere[4]).

The data has then be transferred into Van't Hoff plots for different coverages (Supplementary Figure 3). The isosteric heat of adsorption is defined as the negative of the isosteric enthalpy of adsorption by the relation ($\Delta Q = -\Delta H$).[5] For a given surface coverage, it can be derived from the so-called van´t Hoff form (Eq. 1), which is analogous to the Clausius-Clapeyron equation. A detailed derivation is given elsewhere.[6]

$$\Delta H = R \cdot \left(\frac{\partial \ln(P_{eq})}{\partial \frac{1}{T}} \right)_{n_a} \tag{1}$$

Using a linear interpolation in the adsorption isotherms for different temperatures, we calculate the pressure of equilibrium (P_{eq}) for a fixed amount of adsorbed gas (n_a). The variation of the pressure of equilibrium with the temperature for a fixed amount of gas is the so-called *Isoster* curve and a linear relation is found between the $ln(P_{eq})$ and the reciprocal temperature $1/T$ (see Supplementary Figure 3). A linear regression model was employed for the isosters at different gas concentrations. Using the Equation 1 with the obtained slopes we calculate the isosteric heat of adsorption in function of the coverage (see Supplementary Figure 4).

An alternative analytical approach is based on the Langmuir model. In the low-pressure range we can restrict our considerations to the strong adsorption sites. The data can be fitted to the linear form of the Langmuir equation (Eq. 2).

$$\mathrm{n}_a = \frac{\mathrm{n_m kP}}{1+\mathrm{kP}} \tag{2}$$

Supplementary Figure 2 shows the H_2 and D_2 adsorption isotherms for different temperatures and their corresponding Langmuir fitting using the range (0-0.1bar). Supplementary Table 1 shows the equilibrium constants and saturation volumes for the H_2 and D_2 adsorption isotherms using the Langmuir fitting. The adsorption enthalpies and entropies are obtained by fitting temperature-dependent equilibrium constants (k) to the van't Hoff equation $ln\, k = -\frac{\Delta H}{RT} + \frac{\Delta S}{R}$. The linear fittings for the case of H_2 and D_2 are shown in Supplementary Figure 5.

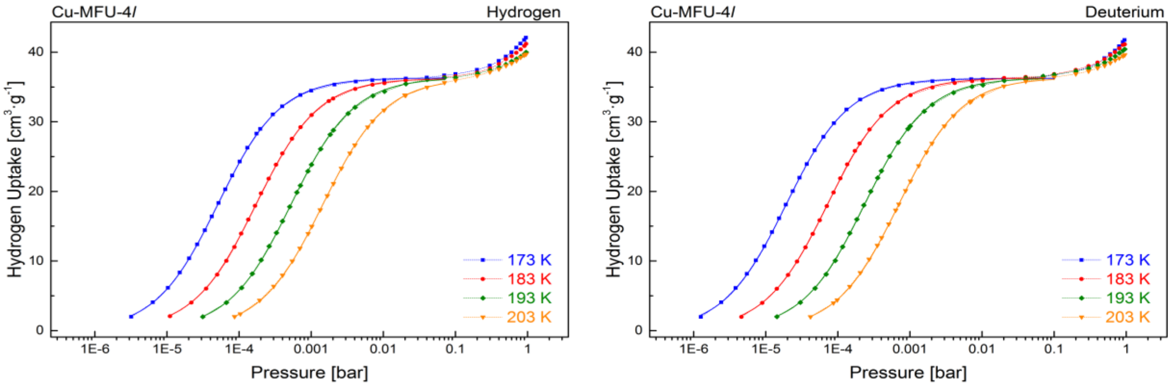

Supplementary Figure 2: Hydrogen (left) and deuterium (right) adsorption isotherms on Cu(I)-MFU-4*l* for different temperatures. The H_2 and D_2 adsorption isotherms show well-separated strong and weak adsorption sites. Cu(I) sites are saturated at a pressure of approx. 0.1 bar.

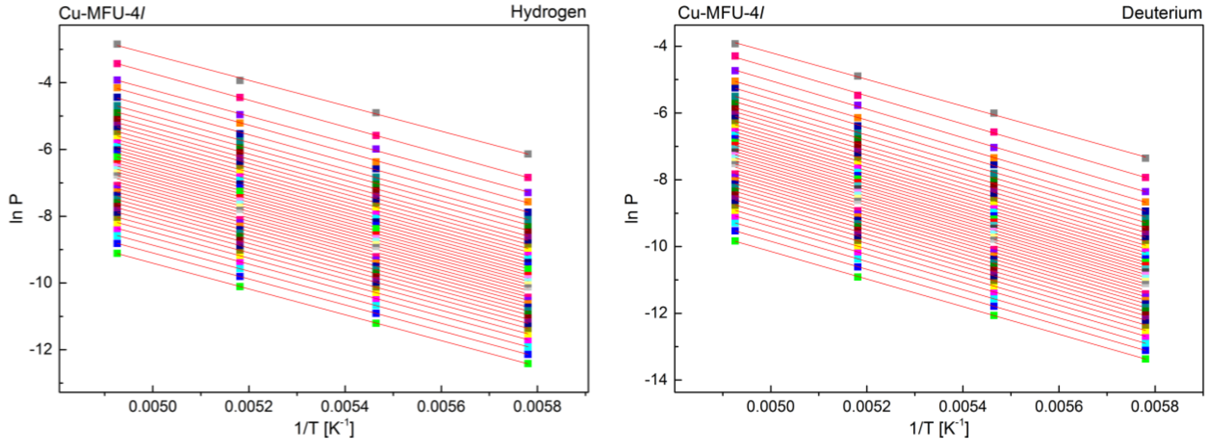

Supplementary Figure 3: Adsorption isosteric method- Van't Hoff plot. Hydrogen (left) and deuterium (right) van't Hoff plot for different coverages.

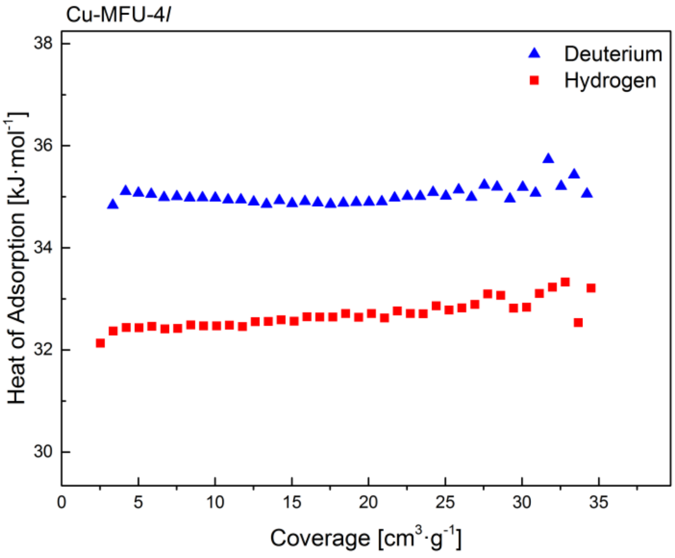

Supplementary Figure 4: Isosteric heat of adsorption. Hydrogen and deuterium isosteric heat of adsorption in function of the coverage calculated using the isosteric method.

Supplementary Table 1: Equilibrium constants and saturation volumes for H_2 and D_2 adsorption on Cu(I)-MFU-4*l*.

T [K]	H_2		D_2	
	k [bar^{-1}]	V_m [cm^3g^{-1}]	k [bar^{-1}]	V_m [cm^3g^{-1}]
173	19690	36.32	52174	36.26
183	5832	36.25	13899	36.32
193	1898	36.34	4252	36.30
203	691	36.23	1423	36.34

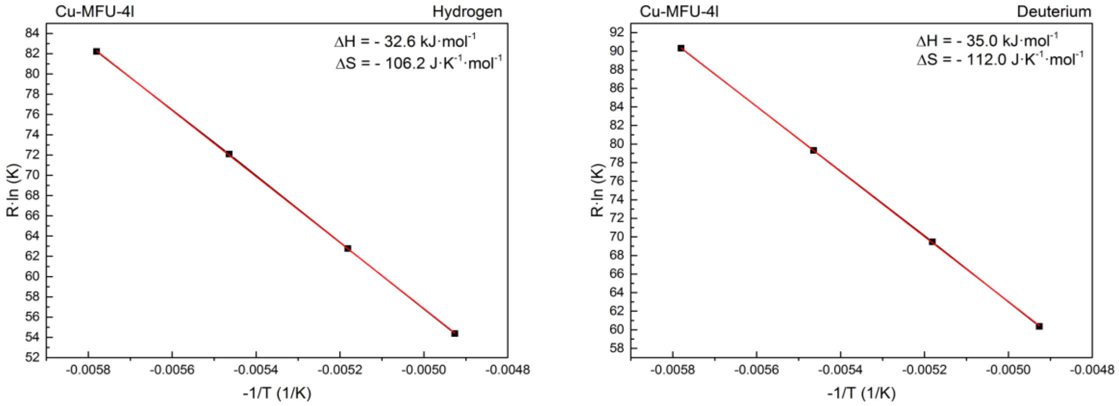

Supplementary Figure 5: Van't Hoff linear fittings using Langmuir model. Hydrogen (left) and deuterium (right) van't Hoff linear fittings using the Langmuir equilibrium constants in the pressure range 0-0.1 bar. The average enthalpy and entropy are calculated using linear regression parameters.

Supplementary Table 2: Average isosteric heat of adsorption for H_2 and D_2 on Cu(I)-MFU-4*l*. The data is obtained from the experimental adsorption isotherms using the isosteric method. Isosteric enthalpy and entropy of adsorption calculated by the Langmuir analytical approach. Errors for the isosteric method correspond to the standard deviations calculated from the average. Errors for the analytical approach correspond to the standard deviations calculated for the linear regression.

Adsorbate	ΔQ [$kJ\cdot mol^{-1}$]	ΔH [$kJ\cdot mol^{-1}$]	ΔS [$J\cdot mol^{-1}\cdot K^{-1}$]
	Isosteric method	Analytical approach	Analytical approach
H_2	32.7 ± 0.3	-32.6 ± 0.2	-105.6 ± 0.9
D_2	35.0 ± 0.3	-35.0 ± 0.1	-111.7 ± 0.9

Supplementary Note 2: Thermal desorption spectroscopy (TDS). TDS is commonly used in surface science to measure the activation energy of desorption. The activation energies can be determined by two methods (see below), utilizing measurements with different heating rates. Both methods are equivalent, do not require any assumption about pre-exponential factor, reaction order or specific mechanism, and the values for the desorption energies are typically in agreement with values for the isosteric heat of adsorption.[7]

After exposure to an equimolar mixture of 10mbar H_2/D_2 at 100K the thermal desorption spectra have been recorded applying different heating rates of $0.01K.s^{-1}$, $0.05K.s^{-1}$and $0.1K.s^{-1}$. The desorption maxima for both H_2 and D_2 show a clear shift to lower temperatures for slower heating rates indicating a thermally activated desorption process (Supplementary Figure 6 left). Owing to the exchange of H_2 by D_2 at an exposure temperature of 100K, the D_2 desorption rate is by an order of magnitude higher than for H_2.

For an adsorbed gas molecule, the desorption process can be described by the Polanyi-Wigner equation for first order with the assumption that the pre-exponential factor and the desorption energy are independent of coverage:

$$-\frac{d\theta}{dt} = \nu \cdot exp\left(-\frac{E_{des}}{RT}\right) \cdot \theta \qquad (3)$$

with instantaneous coverage θ, frequency factor ν, desorption energy E_{des}, gas constant R, and temperature T. In the case of a linear heating rate β, the temperature can be described by

$$T = T_0 + \beta \cdot t \qquad (4)$$

where T_0 is the initial temperature. The Polanyi-Wigner equation can be rewritten to

$$ln\left(\frac{\beta}{T_{max}^2}\right) = -\frac{E_{des}}{RT_{max}} + ln\left(\frac{\nu R \theta_0}{E_{des}}\right) \qquad (5)$$

A plot of $ln\left(\frac{\beta}{T_{max}^2}\right)$ versus $\left(\frac{1}{T_{max}}\right)$ yields a straight line and the desorption energy can be extracted from the slope (see Supplementary Figure 6 right).[8]

Alternatively, Falconer proposed that for the maximum desorption rate at the peak temperature the following relation holds according to equation (3):[7]

$$ln\left(-\frac{d\theta}{dt}\bigg|_{max}\right) = -\frac{E_{des}}{RT_{max}} + ln(\nu \cdot \theta) \qquad (6)$$

Again, the logarithm of the maximum desorption rate versus $\left(\frac{\mathbf{1}}{\boldsymbol{T_{max}}}\right)$ yields a straight line, and the desorption energy can be extracted from the slope (see Supplementary Figure 6 right).

Supplementary Table 3 gives the desorption energies determined by the two independent methods together with the error of the linear regression. For TDS the error including all experimental uncertainties is difficult to quantify, however, can be approximated to about ±2 $kJ.mol^{-1}$. Owing to the smaller signal of H_2 the uncertainty of the evaluation maybe even higher for H_2.

The heats of adsorption and the activation energies of desorption obtained by three different methods are compared in Supplementary Table 5. Here possible experimental uncertainties, which are higher than the standard deviation, are included. All methods are in good agreement within the expected error range. Furthermore, the value for D_2 is about 2.5 $kJ \cdot mol^{-1}$ higher than for H_2 which can be related to the different zero-point energies of H_2 and D_2.

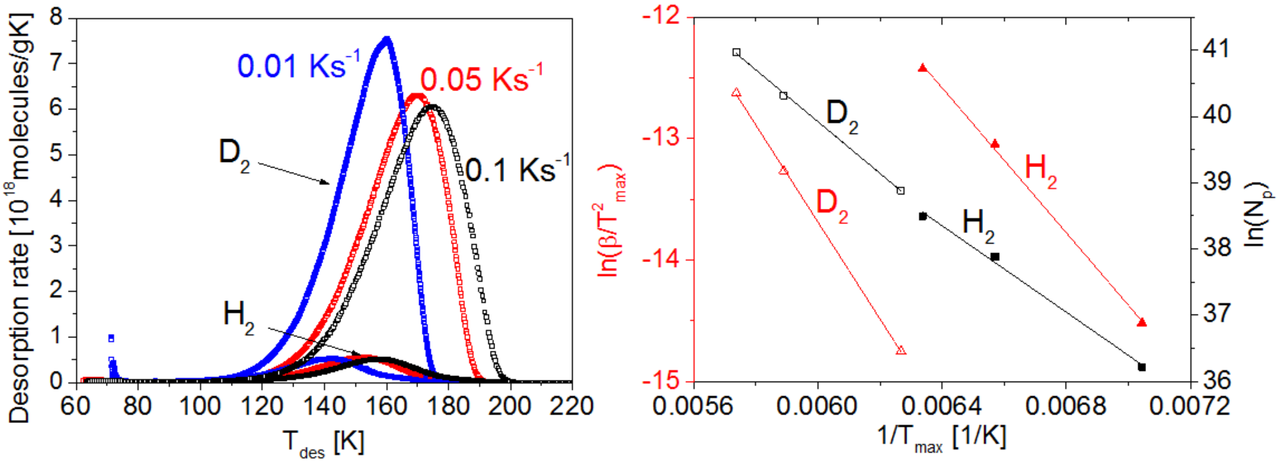

Supplementary Figure 6: TDS spectra of different heating rates. Comparison of different heating rates. Left: The equimolar mixture of 10mbar has been applied for 10min at 100K. The heating procedure has been performed with different heating rates (blue: $0.01 K.s^{-1}$, red: $0.05 K.s^{-1}$, black: $0.1 K.s^{-1}$). Right: Procedure used for the determination of the desorption energy.

Supplementary Table 3: Desorption energies for H_2 and D_2 determined from the TDS measurements by applying different heating rates. Errors for the thermal desorption correspond to the standard deviation calculated for the linear regression.

Adsorbate	E_{des} [kJ.mol^{-1}] from eq. (5)	E_{des} [kJ.mol^{-1}] from eq. (6)
H_2	24.8 ± 0.90	27.1 ± 1.82
D_2	33.2 ± 0.52	32.5 ± 0.93

Supplementary Table 4. Calculated enthalpy and entropy for H_2 and D_2 adsorption on Cu(I)-MFU-4*l*. DFT-D3 calculations following the details laid out in the Methods section have been employed to calculate the enthalpy and entropy of adsorption at the Cu(I) sites of Cu(I)-MFU-4*l*. Note that the in-plane rotation of the adsorbed molecule has been approximated as free rotation.

Adsorbate	ΔH [kJ·mol^{-1}]	ΔS [J·mol^{-1}·K^{-1}]
H_2	-31.2	-100.4
D_2	-34.4	-105.5

Supplementary Table 5. Heat of adsorption and activation energies of desorption obtained by the isosteric method, Langmuir analytical approach, TDS and DFT-D3.

Adsorbate	ΔQ [kJ mol^{-1}]	ΔH [kJ mol^{-1}]	E_{des}[kJ mol^{-1}]	E_{des}[kJ mol^{-1}]	ΔH [kJ mol^{-1}]
	Isosteric method	Analytical Approach	from TDS using eq. (5)	from TDS using eq. (6)	First principles DFT
H_2	32.7 ± 1.0	-32.6 ± 1.0	24.8 ± 2.0	27.1 ± 2.0	-31.2
D_2	35.0 ± 1.0	-35.0 ± 1.0	33.2 ± 2.0	32.5 ± 2.0	-34.4

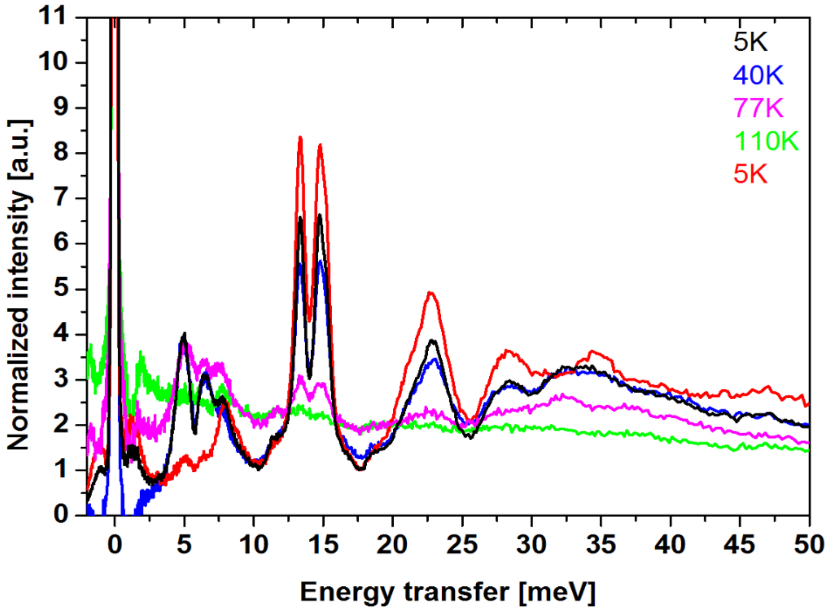

Supplementary Figure 7: Higher-energy signals in the INS spectra recorded at different temperatures. Development of D_2 replacement at the strong adsorption site, during heating and cooling. The peaks at 4.9 and 6.5 meV, which belong to the strong adsorption sites, show no change between the first 5 K (black) and higher temperatures 40 K, 77 K, indicating that the same H_2 amount is still bound to this site. At the weaker adsorption site (13.4 and 14.7 meV) there is already a decrease in intensity for the 40 K measurement. At 77 K, in addition to the peaks of the strong adsorption site, a third peak emerges around 7.9 meV. This 7.9meV peak was hidden in the broader 6.5 meV signal (Figure 3a, high loading) and appears clearly after the exchange of H_2 by D_2 at the strong adsorption site. A comparison of the spectra measured at 5 K before (black) and after (red) heating to 200 K shows that the intensity does not change significantly for the 7.9 meV transition. The 7.9 meV signal can thus be assigned to an orthohydrogen vibration, which is bound to the weaker adsorption site. The peak at 23.0 meV is most likely the combination of a phonon mode and the rotational transition at 14.7 meV. Any transition from the ground state to an excited state of parahydrogen has very low cross section for neutrons,[9] however, the transitions between the ground state to orthohydrogen states have the larger cross section (82 barn) and the combination of a rotational transition and a phonon also has a high cross section. The signal at 28.7 meV belongs to the transition (J=1 to J=2) in weakly adsorbed H_2 due to the presence of a small amount of orthohydrogen. The excess signal that is observed at the higher loading corresponds to the recoil of the molecular hydrogen adsorbed on the second site.[1] The lack of recoil observed in the initial low loading reflects that all hydrogen molecules are bound at the very strong first adsorption site.

Supplementary Note 3: Raman measurements. The 514.5 nm line of an Argon ion laser was used as excitation source with an incident power of less than 5 mW to avoid sample degradation and heating effects. The scattered light was analyzed by a T64000 Jobin-Yvon Raman spectrometer equipped with a liquid-nitrogen-cooled charge coupled detector (CCD). The measurements were performed in the single grating configuration yielding a resolution of 1.9 cm^{-1} in the spectral region of interest. A calibration spectrum of an Ar lamp was recorded after each measurement.

For the application of high gas pressures and low temperatures we used a sample cell specially designed for optical measurements, which is described in reference.[10] After activating at 453 K for 3 h under high vacuum, the sample was loaded in the pressure cell under argon atmosphere in a glove box. Then the cell was connected to a turbo molecular pump, providing high vacuum prior to the gas loading. Only half of the cell was filled with Cu(I)-MFU-4*l* enabling to record reference spectra of the free gas occupying the empty part of the cell under the same pressure/temperature conditions as the sample. The gas pressure inside the cell was stepwise increased up to 22 bar. For the low temperature measurements (down to 40 K), the cell was immersed to a helium bath cryostat with a silicon-diode temperature sensor.

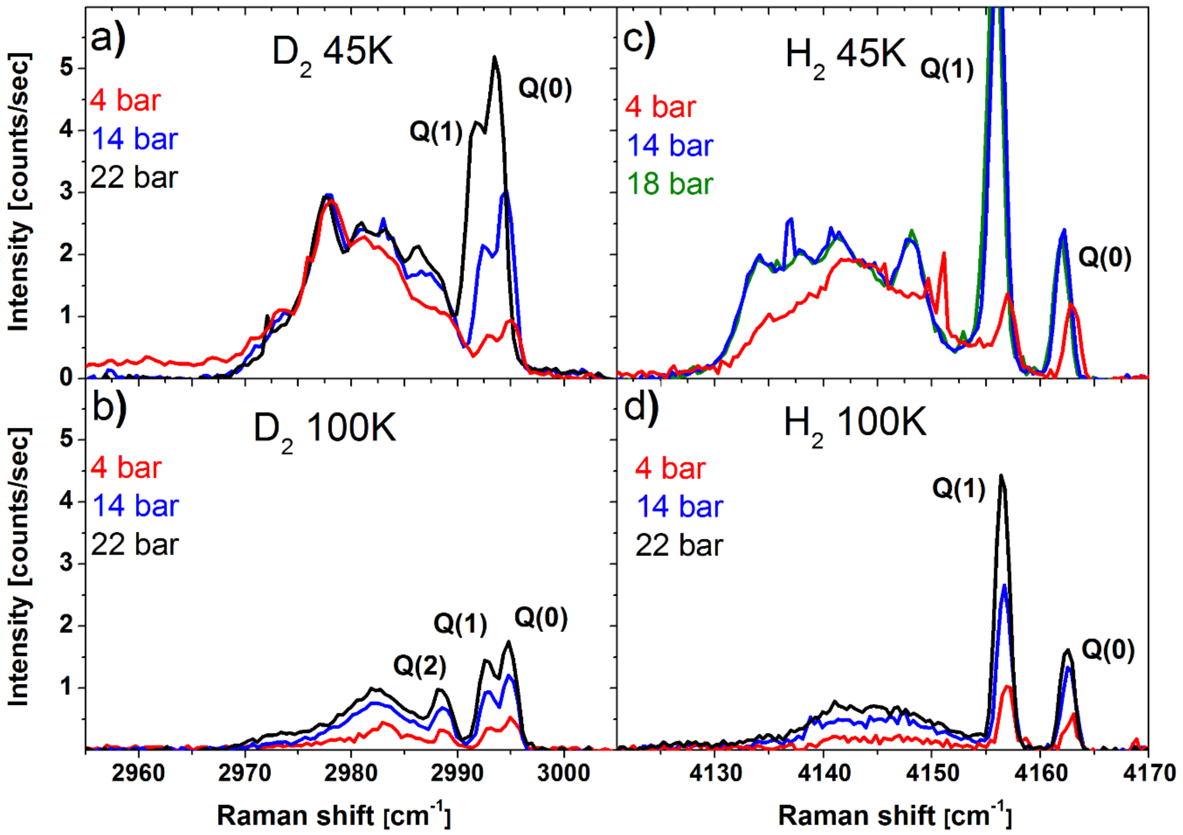

Supplementary Figure 8: Raman spectra of D_2 and H_2. Raman spectra of D_2 (a, b) and H_2 (c, d) at different temperatures and different pressures in Cu(I)-MFU-4*l*. The spectra have been recorded at 45K (a, c) and 100K (b, d) for different pressures. In addition to the Q-lines of the free gases (labeled Q(0), Q(1) and Q(2)) a redshifted signal is observed, which is almost independent of pressure at 45K, whereas at 100K the shifted signal is much weaker and only visible at higher pressures. The attractive interaction between the adsorption sites of Cu(I)-MFU-4*l* and the H_2 (D_2) molecules reduces the degrees of freedom of these physisorbed molecules, which leads to a modified vibrational transitions of the Q(J)-branch.[11]

Supplementary Note 4: Benchmark of DFT calculation protocol. A small molecule (CuH), which represents the Cu(I) site, has been used as a benchmark for the various methods (PBE0-D3, MP2 and CCSD(T)) and basis sets (def2-TZVP, cc-pVTZ, cc-pVQZ) used. We want also to examine the effect of an all-electron (def2-TZVP and cc-pVQZ) versus a basis set with ECP (cc-pVQZ-PP). Moreover, the effect of anharmonicities on frequencies is explored. H_2 is interacting with CuH with a T-shaped orientation, exactly in the manner that is adsorbed on the copper site of the Cu(I)-MFU-4*l*. Results are summarized in Supplementary Table 6. According to Supplementary Table 6, MP2 is overestimating the CCSD(T) binding energies. PBE-D3 are overestimating the CCSD(T) results, whereas PBE0-D3 are closer.

Two models of increasing size are used for the Cu(I)-MFU-4*l*. In the small model (I), termination is done with triazolate anions, whereas the larger model (II) is further extended by a benzene ring. Both models are shown in Supplementary Figure 9. First a benchmark of the model has been done, where it has been investigated if the formate $[HCOO]^-$ anions, which are used to cap the Zn centers, can be replaced by Cl- anions that are easier to model, because of less degrees of freedom in the calculations. In the simpler model (denoted as I), the metal core is capped with triazolate anions. We used two variations for capping the Zn atoms: a) Formate anions $[HCOO]^-$ and b) Cl^- anions. We denote them as I-Form or I-Cl, depending how they are capped. In the second model (denoted as II), the triazolate anions are further extended by a benzene ring. For these comparisons, we use the PBE-D3/def2-TZVP and PBE0-D3/def2-TZVP methods. The results are summarized in Supplementary Table 7. The binding energies and enthalpies change less by 1kJ mol^{-1} between the models I-Form and I-Cl and the frequencies by ~10 cm^{-1}. This validates our approach to use Cl anions instead of formate as capping ligands to the Zn sites.

The second benchmark is on the effect of the model size. We compare results for models I-Cl and II-Cl from the PBE-D3/def2-TZVP and PBE0-D3/def2-TZVP methods in Supplementary Table 8. The results change by -2 and -1 kJ mol^{-1} respectively for H_2 and N_2, when the model size is increased from I to II.

Supplementary Table 6. Computed frequencies (cm^{-1}) and binding energies (BE, in kJ mol^{-1}) from DFT (PBE0-D3 and PBE-D3) versus MP2 and CCSD(T) calculations for the interaction of H_2 with the CuH molecule.

	Free H_2		Adsorb H_2		Δv(ads)		BE
	Harm	Anharm	Harm	Anharm	Harm	Anharm	
CCSD(T)/<br>cc-pVQZ and cc-pVQZ-PP (Cu)	4404	--	3778	--	-626	--	-52.1
CCSD(T)/cc-pVQZ	4404	--	3861	--	-543	--	-46.1
MP2/<br>cc-pVQZ and cc-pVQZ-PP (Cu)	4520	4292	3622	3398	-898	-894	-71.8
MP2<br>aug-ccpVTZ and aug-cc-pVTZ-PP (Cu)	4518	4283	3618	3392	-900	-891	-71.9
PBE0-D3<br>def2-TZVP and aug-cc-pVTZ-PP (Cu)	4415	4190	3781	3524	-634	-666	-51.7
PBE0-D3<br>def2-TZVP and cc-pVTZ-PP (Cu)	4415	4190	3785	3523	-630	-667	-50.3
PBE0-D3/def2-TZVP	4415	4190	3863	3616	-552	-574	-44.5
PBE0-D3<br>def2-TZVPP (H_2), aug-cc-pVTZ-PP (Cu) and def2-TZVP (other atoms)	4408	4181	3764	3511	-644	-670	-53.4
PBE-D3<br>def2-TZVPP (H2), aug-cc-pVTZ-PP (Cu) and def2-TZVP (other atoms)	4317	4084	3525	3257	-792	827	-59.0
PBE-D3<br>def2-TZVP and aug-cc-pVTZ-PP (Cu)	4321	4089	3541	3267	-780	-822	-57.5
PBE-D3<br>def2-TZVP and cc-pVTZ-PP (Cu)	4321	4089	3536	3257	-785	-832	-57.0
PBE-D3/def2-TZVP	4321	4089	3623	3324	-689	-765	-53.1

Binding energies are corrected for the BSSE and given in kJ mol^{-1}. Frequencies are in cm^{-1}.

Supplementary Table 7. Effect of the Zn-capping ligands (formate versus chloride anions) on the calculated binding energies (ΔE, kJ.mol^{-1}), enthalpies (ΔH, kJ.mol^{-1}) and frequencies (Δv, cm^{-1}) of N_2, H_2 and CO adsorbed on the Cu(I) sites. Comparison with available experimental results is also done. Models are shown in Supplementary Figure 9.

	model	I-Form			I-Cl		
		ΔE	ΔH(177K)	Δv	ΔE	ΔH(177K)	Δv
N_2	PBE-D3/def2-TZVP	-71.3	-66.7	-137	-70.4	-65.8	-135
	PBE0-D3/def2-TZVP	-48.6	-44.7	-87	-48.3	-44.5	-83
	Experimental		-42	-89		-42	-89
H_2	PBE-D3/def2-TZVP	-40.3	-32.3	-992	-40.9	-33.0	-977
	PBE0-D3/def2-TZVP	-35.4	-27.4	-789	-36.3	-28.3	-779
	Experimental		-32			-32	
CO	PBE-D3/def2-TZVP	-140.7			-139.5	-133.2	-66
	PBE0-D3/def2-TZVP	-113.2	-107.5	-50	-112.5	-106.8	-45
	Experimental			-62			-62

Supplementary Table 8. Effect of model size on the binding energies (ΔE, kJ mol^{-1}), enthalpies (ΔH, kJ mol^{-1}) and frequencies (Δv, cm^{-1}) of N_2, H_2, CO and CH_4 adsorbed on Cu(I) sites. Energies are in kJ mol^{-1} and frequencies in cm^{-1}. Comparison with available experimental results is also done. Models are shown in Supplementary Figure 9.

	model	I-Cl			II-Cl		
		ΔE	ΔH(177K)	Δv	ΔE	ΔH(177K)	Δv
N_2	PBE-D3/def2-TZVP	-70.4	-65.8	-135	-70.1	-65.5	-141
	PBE0-D3/def2-TZVP	-48.3	-44.5	-83	-49.2	-45.2	-88
	Experimental		-42	-89		-42	-89
H_2	PBE-D3/def2-TZVP	-40.9	-33.0	-977	-42.9	-35.0	-989
	PBE0-D3/def2-TZVP	-36.3	-28.3	-779	-38.8	-30.8	-804
	Experimental		-32			-32	
CO	PBE-D3/def2-TZVP	-139.5	-133.2	-66	-143.7	-137.4	-76
	PBE0-D3/def2-TZVP	-112.5	-106.8	-45	-117.6	-111.8	-54
	Experimental			-62			-62
CH_4	PBE-D3/def2-TZVP	-19.0	-17.3		-21.7		
	PBE0-D3/def2-TZVP	-19.2	-17.0		-22.5	-20.1	
	Experimental		-15			-15	

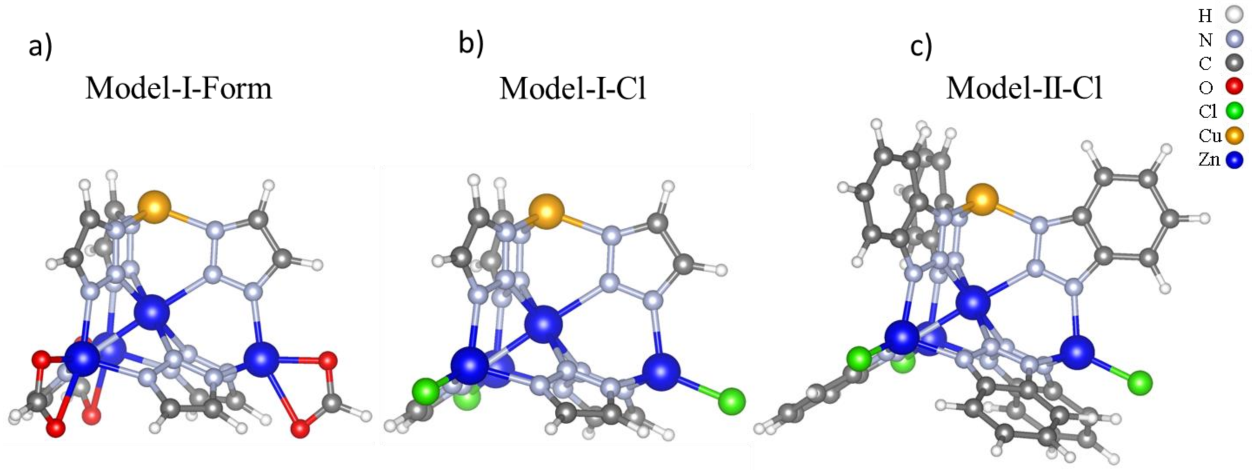

Supplementary Figure 9: Atomistic models for the first principles calculations. Models used to represent the SBU of the Cu(I)-MFU-4*l*: Model-I-Form (**a**), Model-I-Cl (**b**) and Model-II-Cl (**c**). Colour scheme: white – H, light blue – N, gray – C, red – O, green – Cl, orange – Cu, dark blue – Zn.

Supplementary Note 5: Error estimate for using molecular instead of periodic model. We performed further calculations to assess the error in the computed binding energies of H_2, from using a finite cluster model instead of the periodic cell. Due to the big cell size, geometry optimization is computationally time and resource demanding, thus an approximate scheme has been used by taking the crystal structure of the original MFU-4*l* and replacing one of the units in the corner with the atomic positions of the Model-II-Cl (shown in Supplementary Figure 9). We used the PBE0-D3 optimized coordinates of the Model-II-Cl with H_2. Then three single point energy calculations are performed: dimer of H_2@Cu-MFU-4*l*, Cu-MFU-4*l* and H_2 at the positions of the dimer. In this case, the interaction energies are calculated rather than the binding energies, i.e. the deformation energies of the individual monomers are not included. That is the reason, why the interaction energies are much higher compared to binding energies. These periodic calculations have been performed with the VASP 5.2 program and using the PBE functional with the –D3 dispersion corrections and 400eV kinetic energy cutoff. We have also used the dispersion parameters derived for the PBE0 functional. The same procedure was employed for the molecular Model-II-Cl inserted in a cubic cell of 25.0Å. In this way, we can estimate the long-range effects of the H_2 binding in the crystal. We further decompose the interaction energies into contributions from PBE and –D3 dispersions only. The results are shown in Supplementary Table 9. These numbers cannot be directly compared to the PBE values of Supplementary Table 8, because the deformation energies are not included. However, we can get useful conclusions about the DFT error with respect to the model. Our finite molecular model has an approximate error of ~1 kJ mol^{-1}. The long-range dispersions are estimated to be 0.2 kJ mol^{-1}, irrespective of the PBE or PBE0 dispersion parameters. The long-range correction from the DFT is estimated to be 0.5kJ mol^{-1}. This gives a final estimate value of 0.7 kJ mol^{-1} for the error introduced by using molecular model.

Additionally, the same process is done to estimate the difference in the H_2 rotational barriers. The results are shown in Supplementary Figure 10. The barriers are identical, thus the long-range corrections play no role for the rotation of H_2 around the Cu(I) site. Furthermore, we found that the barrier gets slightly larger, when the H-H gets elongated.

Supplementary Table 9. Long range corrections for the H_2 binding with the Cu(I) site.

Models	PBE	-D3 (PBE)	-D3 (PBE0)	
Periodic	-67.5	-6.5	-6.2	
Model-II-Cl	-67.0	-6.3	-6.0	
				Total
Long-Range corrections	-0.5	-0.2	-0.2	-0.7

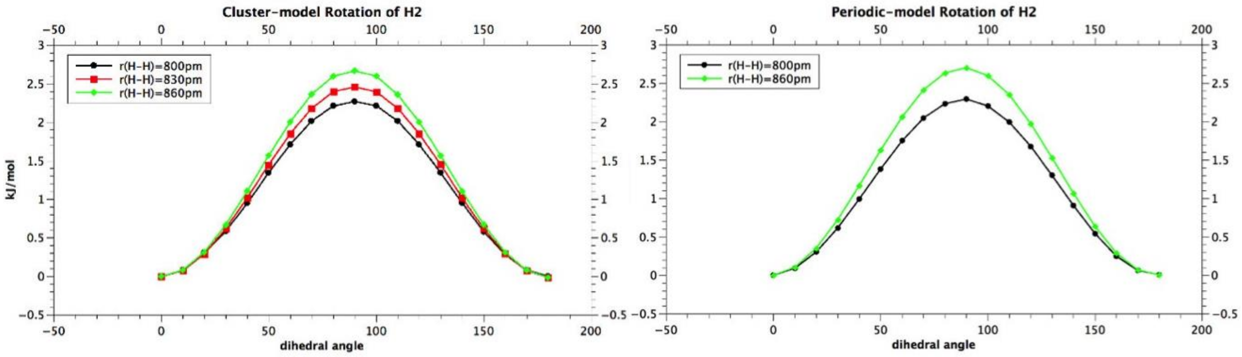

Supplementary Figure 10: Potential energy surface of rotating H_2. Rotational barrier of H_2 in (left) molecular Model-II-Cl and (right) periodic structure of Cu(I)-MFU-4*l*. Three different bond lengths for H_2 are considered.

Supplementary Note 6: Energy Decomposition Analysis.

The energy decomposition analysis (EDA),[12] as implemented in the ADF[13] program, was used to analyze the interactions between H_2 and the Cu(I) site. In this scheme, the binding energy can be written as the sum of the interaction energies (ΔE_{int}) and the deformation energies (ΔE_{def}) of the monomers. ΔE_{int} is further decomposed into terms that contain a) the electrostatic interactions (ΔV_{elst}) between the unperturbed charge distributions of the deformed fragment with the field of the other, b) the Pauli repulsion (ΔE_{Pauli}), which is associated with going from the unperturbed individual fragments to a symmetrized and orthonormalized wave function of their product $\Psi_0 = N\mathbf{A}[\Psi_A\Psi_B]$ that obeys the Pauli exclusion principle (N is a normalization constant and **A** the anti-symmetry operator), and c) orbital interactions (ΔE_{OI}) that account for electron pair bonding, charge transfer, and polarization effects when going from Ψ_0 to the converged wave function of the complex. The first two terms can be combined and the sum of the electrostatic interactions and Pauli repulsion is called steric interaction. The orbital interactions can be further decomposed by employing the natural orbitals for chemical valence (NOCV) theory in combination with the extended transition state (ETS) method.[14,15] The ETS-NOCV decomposes the orbital interactions into different components (σ, π, δ) of the chemical bond. These calculations have been performed with the ADF program using similar computational details as before, that is, the PBE0-D3 functional and the TZP basis set. The results are presented in Supplementary Table10.

According to the EDA, the electrostatic interactions are 62% of the total attractive interactions, which is the sum of the electrostatic, the orbital interactions and the dispersions. Dispersions are very small, only 2%, and orbital interactions reach 36%. The orbital interactions are further decomposed with the ETS-NOCV scheme. After analyzing the NOCVs, we identified the pair of NOCVs that contribute to forward- and back-donation. Contour plots of the most important NOCVs are shown in Supplementary Figure 11. According to the ETS-NOCV, forward-donation terms are 63.5 kJ mol^{-1} or 62% of the total orbital interactions. Back-donation terms contribute a significant amount of 34.9 kJ mol^{-1} or 34%.

Supplementary Table 11 shows calculated expectation values of adsorbed H_2 and D_2 as calculated from a Morse fit. It further includes thermodynamic data based on the six

characteristic modes that are assigned according to motion in free space (rotation R, translation T).

Supplementary Table 10. Energy decomposition analysis (EDA) from ADF. Decomposition of Orbital Interaction into Forward- and Back-donation terms. All energies are in kJ mol^{-1}. Values in parentheses refer to % contribution to the total attractive interactions (ΔV_{elst} + ΔE_{OI} + Dispersion). Abbreviations: ΔV_{elst} = electrostatic interactions, ΔE_{Pauli} = Pauli repulsion, and ΔE_{OI} = orbital interactions. All energies are in kJ mol^{-1}.

H_2	Total bonding	ΔV_{elst}	ΔE_{Pauli}	ΔE_{OI}	Dispersion
	-53.2	-172.8 (62%)	226.0	-102.1 (36%)	-4.4 (2%)
ΔE_{OI}	Forward-donation (σ H_2 → M)			-63.5 (62%)	
	Back-donation (M → σ* H_2)			-34.9 (34%)	

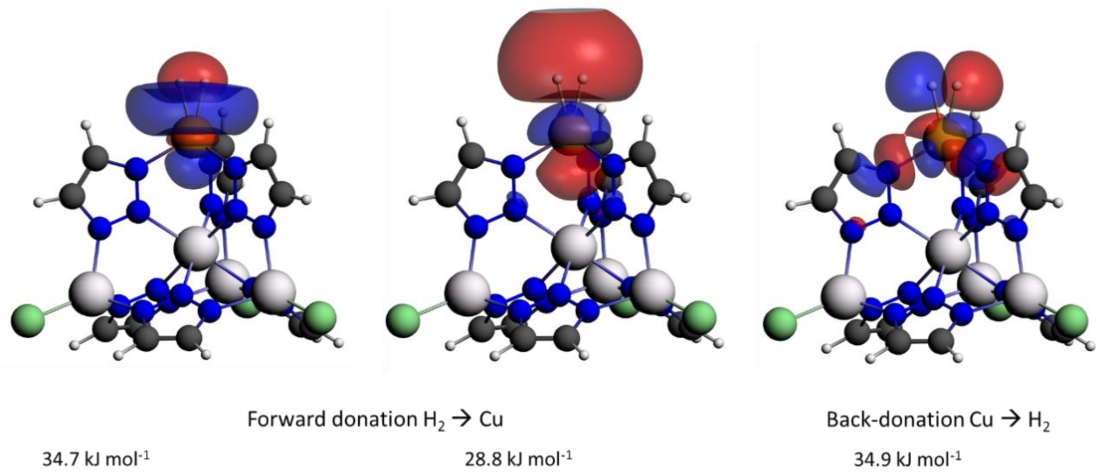

Supplementary Figure 11: Natural orbitals of adsorbed H_2. Contour plots of NOCVs that contribute to the orbital interactions as calculated from the ETS-NOCV scheme.

Supplementary Table 11. Expectation value $< \mathrm{R} >$ of the distance between Cu and the centre of the adsorbed H_2 or D_2 isotopologues; expectation values $< \mathrm{r} >$, of bond length H-H or D-D of adsorbed hydrogen isotopologues; binding energy of adsorbed H_2 on the Cu(I) site($\Delta \mathrm{E}_{\mathrm{pot}}$); zero-point energy of adsorbed H_2 and D_2 ($\mathrm{E}_{\mathrm{ZPE}}$); Entropy of adsorbed H_2 and D_2 at 77K (ΔS); Enthalpy of adsorbed H_2 and D_2 at 77K (ΔH); Gibbs energy of adsorbed H_2 and D_2 at 77K (ΔG); frequencies of adsorbed H_2 (ν). The calculations have been done with PBE0-D3/def2-TZVPP (H_2)/aug-cc-pVTZ-PP (Cu)/def2-TZVP (other atoms) on the model-I-Cl of Cu(I)-MFU-4*l* (Supplementary Figure 9b).

Hydrogen isotopologue	$< \mathrm{R} >$ [Å]	$< \mathrm{r} >$ [Å]	$\Delta \mathrm{E}_{\mathrm{pot}}$ [kJ ·mol^{-1}]	$\Delta \mathrm{E}_{\mathrm{ZPE}}$ [kJ ·mol^{-1}]	ΔS [J mol^{-1} ·K^{-1}]	ΔH [kJ ·mol^{-1}]	ΔG [kJ ·mol^{-1}]	ν [cm^{-1}]
Adsorbed H_2	1.738	0.830	-41.1	11.1	-100.4	-31.2	-27.3	283R, 315T, 901T, 901T, 1297R, 3481
Adsorbed D_2	1.712	0.824	N/A	7.9	-105.5	-34.4	-24.9	

$\mathrm{E}_{\mathrm{ZPE}}$(free H_2)=26.4 kJ mol^{-1}; $\mathrm{E}_{\mathrm{ZPE}}$(free D_2)=18.6 kJ mol^{-1}; ν(free H_2)=4408 cm^{-1}. $\nu^{R,T}$ corresponds to modes associated to rotations and translations of the free molecules, respectively.

References

1. Mitchell, P. C. H., Parker, S. F., Ramirez-Cuesta, A. J. & Tomkinson, J. *Vibrational spectroscopy with neutrons with applications in chemistry, biology, material science and catalysis*. World Scientific (2005).
2. Clark S. J., *et al.* First principles methods using CASTEP. *Z. Kristallographie* **220**, 567-570 (2005).
3. Ramirez-Cuesta A. J. aCLIMAX 4.0.1, The new version of the software for analyzing and interpreting INS spectra. *Comput. Phys. Commun.* **157**, 226-238 (2004).
4. Kloutse A.F. *et al.* Isosteric heat of hydrogen adsorption on MOFs: comparison between adsorption calorimetry, sorption isosteric method, and analytical models. *Appl. Phys. A*, 1-8 (2015).
5. Sircar, S., Mohr, R., Ristic, C. & Rao, M. B. Isosteric heat of adsorption: Theory and experiment. *J. Phys. Chem. B* **103**, 6539-6546 (1999).
6. Rouquerol, F., Rouquerol, J. & Sing, K. S. W. *Adsorption by powders and porous solids : principles, methodology, and applications*. Academic Press (1999).
7. Falconer, J. L. & Madix, R. J. Flash Desorption Activation-Energies - Dcooh Decomposition and Co Desorption from Ni(110). *Surf. Sci.* **48**, 393-405 (1975).
8. Falconer, J. L. & Schwarz, J. A. Temperature-Programmed Desorption and Reaction - Applications to Supported Catalysts. *Catal. Rev.* **25**, 141-227 (1983).
9. Young, J. A. & Koppel, J.U. Slow Neutron Scattering by Molecular Hydrogen + Deuterium. *Phys. Rev.* **135**, A603-+ (1964).
10. Panella, B., Hirscher, M. & Ludescher, B. Low-temperature thermal-desorption mass spectroscopy applied to investigate the hydrogen adsorption on porous materials. *Microporous Mesoporous Mater.* **103**, 230-234 (2007).
11. Centrone, A., Brambilla, L. & Zerbi, G. Adsorption of H-2 on carbon-based materials: A Raman spectroscopy study. *Phys. Rev. B* **71**, (2005).
12. Bickelhaupt F. M. & Baerends, E.J. In: *Reviews in Computational Chemistry* (ed^(eds Lipkowitz KB, Boyd DD). Jon Wiley and Sons (2007).
13. te Velde, G. *et al.* Chemistry with ADF. *J. Comput. Chem.* **22**, 931-967 (2001).
14. Nalewajski, R. F., Mrozek, J. & Michalak, A. Two-electron valence indices from the Kohn-Sham orbitals. *Int. J. Quantum Chem.* **61**, 589-601 (1997).
15. Michalak, A., DeKock, R. L. & Ziegler, T. Bond multiplicity in transition-metal complexes: Applications of two-electron valence indices. *J. Phys. Chem. A* **112**, 7256-7263 (2008).
